# Dynamin-Like Proteins of Endocytosis in Plants Are Coopted by Potyviruses To Enhance Virus Infection

**DOI:** 10.1128/JVI.01320-18

**Published:** 2018-11-12

**Authors:** Guanwei Wu, Xiaoyan Cui, Hui Chen, Justin B. Renaud, Kangfu Yu, Xin Chen, Aiming Wang

**Affiliations:** a London Research and Development Centre, Agriculture and Agri-Food Canada, London, Ontario, Canada; b Institute of Industrial Crops, Jiangsu Academy of Agricultural Sciences/Jiangsu Key Laboratory for Horticultural Crop Genetic Improvement, Nanjing, Jiangsu, People’s Republic of China; c Department of Biology, University of Western Ontario, London, Ontario, Canada; d Harrow Research and Development Centre, Agriculture and Agri-Food Canada, Harrow, Ontario, Canada; University of Maryland, College Park

**Keywords:** *Arabidopsis*, potyvirus, SMV, TuMV, dynamin, endocytosis, host factor, soybean

## Abstract

Endocytosis and endosomal trafficking regulate the proteins targeted to the plasma membrane and play essential roles in diverse cellular processes, including responses to pathogen attack. Here, we report the identification of Glycine max (soybean) endocytosis dynamin-like protein 5A (GmSDL5A) associated with purified soybean mosaic virus (SMV) virions from soybean using a bottom-up proteomics approach. Knockdown of *GmSDL5A* and its homologous gene *GmSDL12A* inhibits SMV infection in soybean. The role of analogous dynamin-like proteins in potyvirus infection was further confirmed and investigated using the *Arabidopsis*/turnip mosaic virus (TuMV) pathosystem. We demonstrate that dynamin-related proteins 2A and 2B in Arabidopsis thaliana (AtDRP2A, AtDRP2B), homologs of GmSDL5A, are recruited to the virus replication complex (VRC) of TuMV. TuMV infection is inhibited in both A. thaliana *drp2a* (*atdrp2a*) and *atdrp2b* knockout mutants. Overexpression of AtDRP2 promotes TuMV replication and intercellular movement. AtRDP2 interacts with TuMV VPg, CP, CI, and 6K2. Of these viral proteins, VPg, CP, and CI are essential for viral intercellular movement, and 6K2, VPg, and CI are critical components of the VRC. We reveal that VPg and CI are present in the punctate structures labeled by the endocytic tracer FM4-64, suggesting that VPg and CI can be endocytosed. Treatment of plant leaves with a dynamin-specific inhibitor disrupts the delivery of VPg and CI to endocytic structures and suppresses TuMV replication and intercellular movement. Taken together, these data suggest that dynamin-like proteins are novel host factors of potyviruses and that endocytic processes are involved in potyvirus infection.

**IMPORTANCE** It is well known that animal viruses enter host cells via endocytosis, whereas plant viruses require physical assistance, such as human and insect activities, to penetrate the host cell to establish their infection. In this study, we report that the endocytosis pathway is also involved in virus infection in plants. We show that plant potyviruses recruit endocytosis dynamin-like proteins to support their infection. Depletion of them by knockout of the corresponding genes suppresses virus replication, whereas overexpression of them enhances virus replication and intercellular movement. We also demonstrate that the dynamin-like proteins interact with several viral proteins that are essential for virus replication and cell-to-cell movement. We further show that treatment of a dynamin-specific inhibitor disrupts endocytosis and inhibits virus replication and intercellular movement. Therefore, the dynamin-like proteins are novel host factors of potyviruses. The corresponding genes may be manipulated using advanced biotechnology to control potyviral diseases.

## Abstract (precis)

It is well known that animal viruses enter host cells via endocytosis, whereas plant viruses require physical assistance, such as human and insect activities, to penetrate the host cell to establish their infection. In this study, we report that the endocytosis pathway is also involved in virus infection in plants. We show that plant potyviruses recruit endocytosis dynamin-like proteins to support their infection. Depletion of them by knockout of the corresponding genes suppresses virus replication, whereas overexpression of them enhances virus replication and intercellular movement. We also demonstrate that the dynamin-like proteins interact with several viral proteins that are essential for virus replication and cell-to-cell movement. We further show that treatment of a dynamin-specific inhibitor disrupts endocytosis and inhibits virus replication and intercellular movement. Therefore, the dynamin-like proteins are novel host factors of potyviruses. The corresponding genes may be manipulated using advanced biotechnology to control potyviral diseases.

## INTRODUCTION

Viruses are obligate intracellular parasites that infect all living organisms and cause serious diseases worldwide. Different from animal viruses that utilize endocytosis to enter cells, plant viruses initiate their infection through mechanical inoculation or insect-mediated injection to pass through the cell wall barrier. The majority of known plant viruses are positive-sense, single-stranded RNA viruses which have a relatively small genome with limited coding capacity. These RNA viruses typically encode a single-digit number of viral proteins and, thus, must usurp cellular machineries and factors to survive and replicate in their host cells. In the past decade, a large number of host proteins have been identified for viruses to establish their infection in plants (1–3). Many of these host proteins are recruited to form the virus replication complex (VRC) that is associated with modified cellular membranes for the reproduction of their progeny.

Endocytosis involves the internalization or uptake of plasma membrane (PM) proteins or extracellular materials into the cell via a series of vesicle compartments (4). Endocytosis may be clathrin mediated (CME) or clathrin independent. The former is relatively better characterized, whereas the latter remains largely to be understood. As in animal cells, CME is a predominant internalization pathway in plant cells (5) and initiates from the formation of clathrin-coated pits (CCPs), followed by cargo selection. The recognition and recruitment of cargo proteins are mediated by adaptor protein complexes (6). Then, mature clathrin-coated vesicles (CCVs) detach from the PM by dynamin (7) and enter the cytosol, where they are targeted to and fused with the early endosome and recycling endosome *trans*-Golgi network (TGN/EE). Subsequently, these vesicles are recycled back to the PM/cell wall/cell plate via different pathways or delivered into multivesicular bodies, also named the late endosome (LE), which will sort PM proteins to vacuoles for degradation (8, 9).

Dynamin and dynamin-related proteins (DRPs) are multidomain large GTPases that function in many cellular processes underlying membrane dynamics (such as endocytosis) and also act in post-Golgi network clathrin-mediated trafficking in animals (7, 10). Based on the sequences, all dynamin members contain a conserved N-terminal GTP-binding domain, a middle domain that mediates dimerization during self-assembly, and a GTPase-effector domain that modulates GTPase activity. In addition, they have a pleckstrin homology (PH) domain and a C-terminal proline-rich domain (PRD) that interact with Src homology 3 domain-containing proteins, which is critical to the recruitment of dynamin to CCPs (11). Most land plants have six types of DRPs (DRP1 to -6) based on domain structure (12), and only two of them, DRP1 and DRP2, have been reported to participate in CCV formation during endocytosis (13) and post-Golgi network trafficking (14, 15).

In Arabidopsis thaliana, the DRP1 subfamily consists of five members (DRP1A to DRP1E) with a high degree sequence identity (63% to 82%) at the amino acid level (16). The *Arabidopsis* DRP1 homologs lack a PH domain and a PRD and show highly variable and tissue-type expression profiles (17, 18). The DRP2 subfamily in *Arabidopsis* comprises only two members, DRP2A and DRP2B (13). The amino acid sequences of DPR2A and DPR2B are highly identical (93%) and harbor all five domains (13, 17). DPR2A and DPR2B are expressed throughout development in *Arabidopsis* (19). The *drp2a drp2b* double mutant shows gametophyte-lethal phenotypes; however, no loss-of-function phenotypes have been detected in either *drp2a* or *drp2b* mutants, suggesting that their two proteins have some functional redundancy in plant development (19).

Potyviruses account for ∼30% of the currently known plant viruses and include many agriculturally and economically important viruses, such as *Turnip mosaic virus* (TuMV), *Soybean mosaic virus* (SMV), *Plum pox virus* (PPV), and *Potato virus Y* (PVY) (17, 18, 20). Potyviruses have a positive-strand, ∼10-kb RNA genome that encodes a large polyprotein precursor and a small open reading frame (ORF) resulting from RNA polymerase slippage in the P3 coding sequence (17). The two polyproteins are processed co- and posttranslationally by three viral proteases into 11 mature proteins: P1, helper component proteinase (HC-Pro), P3, P3N-pretty interesting *Potyviridae* ORF (P3N-PIPO), 6K1, cylindrical inclusion (CI) protein, 6K2, viral-genome-linked protein (VPg), nuclear inclusion a proteinase (NIa-Pro), nuclear inclusion b (NIb), and capsid protein (CP) (17).

Toward a better understanding of molecular potyvirus-host interactions and identification of novel host factors of potyviruses, we employed gel fractionation and liquid chromatography-tandem mass spectrometry (LC-MS/MS) methods to characterize host proteins associated with SMV virions. We identified an endocytosis protein dynamin, Glycine max (soybean) endocytosis dynamin-like protein 5A (GmSDL5A). We show that this protein interacts with potyviral proteins and is essential for potyvirus infection.

## RESULTS

### Identification of a dynamin-like protein associated with SMV virions.

**FIG 1:**
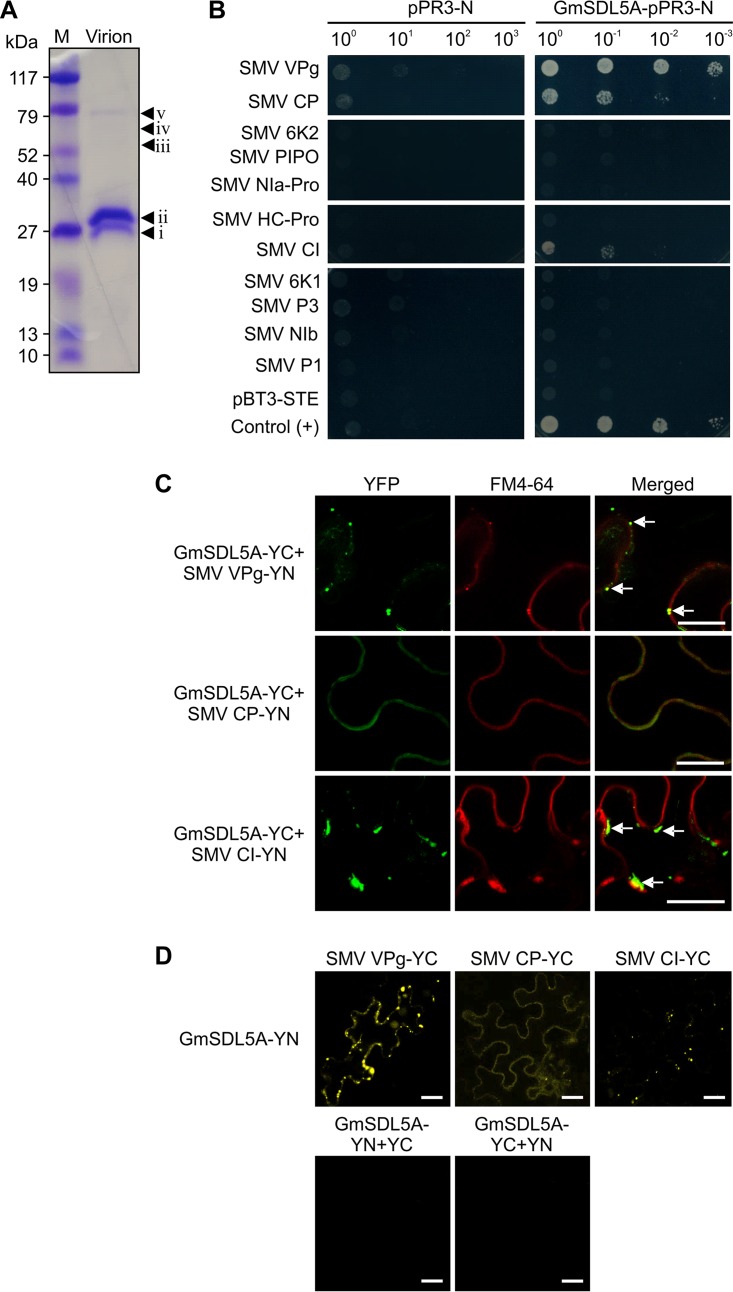
A dynamin-like protein in soybean is associated with *Soybean mosaic virus* virions mediated by SMV capsid components. (A) Analysis of SMV virions purified from infected soybean leaves. The SMV preparation was separated by 10% SDS-PAGE and stained with Coomassie brilliant blue solution. Five individual bands are indicated with arrowheads; they were excised from the gel, digested with trypsin, and analyzed by LC-MS/MS. (B) Protein-protein interaction assay between GmSDL5A and SMV proteins by using a membrane yeast two-hybrid method. Control (+) yeast cells were cotransformed with TuMV 6K2 and AtVAP27-1. (C) BiFC assay of interactions between GmSDL5A and SMV VPg, CP, and CI in *N. benthamiana* cells. The YFP field, FM4-64 dye staining, and overlay of them are shown. Split YFP halves (YN and YC) were fused to the C termini. White arrows indicate the colocalization of the interaction complex of GmSDL5A and SMV proteins, with the endosome compartments labeled by FM4-64. Scale bar = 20 μm. (D) BiFC assay of GmSDL5A and SMV VPg, CP, and CI in *N. benthamiana* cells. YC and YN were exchanged for each pair tested in panel C. Representative negative controls are given. Scale bar = 20 μm.

To isolate the host proteins related to potyvirus infection, we purified the SMV virions from infected soybean leaves. SDS-PAGE analysis of the purified SMV virions visualized five protein bands after Coomassie brilliant blue (CBB) staining (Fig. 1A). Among them, two that were nearly 30 kDa in size, designated bands i and ii, were supposed to be the CP of SMV. We conducted a top-down proteomics analysis to determine their identities and obtain the accurate *m/z* of the intact protein. Results showed that both proteins did correspond to SMV CP (Table 1; see also Table S1 in the supplemental material); however, the measured mass of the CP (29,900 ± 1.9 Da) did not completely match the theoretical mass (29,828 Da).

**TABLE 1:** Viral and host proteins identified by mass spectrometry analysis from purified *Soybean mosaic virus* virions in soybean

Band(s)	Viral/host protein (accession no.)	Coverage(s) (%)	Σ unique peptide(s)	Σ peptide(s)	Molecular mass (Da)
i, ii	CP	55.47, 51.70^*a*^	48, 40^*a*^	48, 40^*a*^	29,828
iv, v	CI	46.85	34	34	71,270
v	Dynamin 5A (Q39828)	19.51	1	9	68,290
iii	Catalase-4	13.79	2	5	56,737
iii, iv	Ribulose bisphosphate carboxylase large chain	14.32	6	6	52,580
iii, iv	K7KRS4_SOYBN uncharacterized protein	13.59	4	6	51,810
iii, iv	I1KJS5_ SOYBN uncharacterized protein	17.83	4	7	48,600

a The values corresponding to both bands i and ii in Fig. 1A are indicated.

The other three protein bands above CP with the sizes between 52 and 79 kDa (Fig. 1A), named bands iii to v, were divided into sections of 1 cm in height. These three gel slices were then subjected to in-gel digestion with trypsin, followed by LC-MS/MS analysis by using a bottom-up proteomics method. The MS analysis identified six unique proteins (Table 1; Table S1). In addition to the SMV protein CI, a total of 33 peptides corresponding to five soybean proteins were identified. Of the nine identified dynamin-related protein 5A (NCBI accession no. Q39828) peptides, eight are shared by another dynamin-related protein, 12A (Q39821), and one is unique to Q39828 (Fig. 1A). These two dynamin-like proteins were first isolated from soybean (21), and their amino acid sequences are highly identical (>98%). Q39828 and Q39821 were designated GmSDL5A and GmSDL12A, respectively.

### GmSDL5A interacts with SMV proteins VPg, CP, and CI.

In addition to CP, three viral proteins, namely, VPg, HC-Pro, and CI, are associated with potyviral virions (22–25). Therefore, we first tested the interaction between GmSDL5A and each of these four SMV proteins. Since dynamin has been reported to be membrane associated (21, 22), we used a split-ubiquitin membrane-based yeast two-hybrid (Y2H) system to probe the potential interactions. *GmSDL5A* and the cDNA fragments coding for the four SMV proteins were cloned into the prey vector pPR3-N and the bait vector pBT3-STE, respectively. All of the NMY51 yeast cells coexpressing the SMV bait proteins with the prey GmSDL5A or empty prey plasmids grew robustly on the double-dropout medium, synthetic defined medium lacking Leu and Trp. Saccharomyces cerevisiae cells cotransfected with the TuMV 6K2 bait and Arabidopsis thaliana VPA27-1 (AtVPA27-1) prey served as a positive control (23). Transformants were first plated on the medium lacking leucine, tryptophan, histidine, and adenine to select for protein interactions on the basis of the activation of the *ADE2* and *HIS3* reporter genes. Background growth due to the leaky expression of *HIS3* was suppressed by adding 3-aminotriazole (3AT). We found that, except for HC-Pro, all three other SMV proteins showed an interaction with GmSDL5A (Fig. 1B). In contrast, none of the seven remaining SMV-encoded proteins interacted with GmSDL5A in the yeast cells (Fig. 1B).

**FIG 2:**
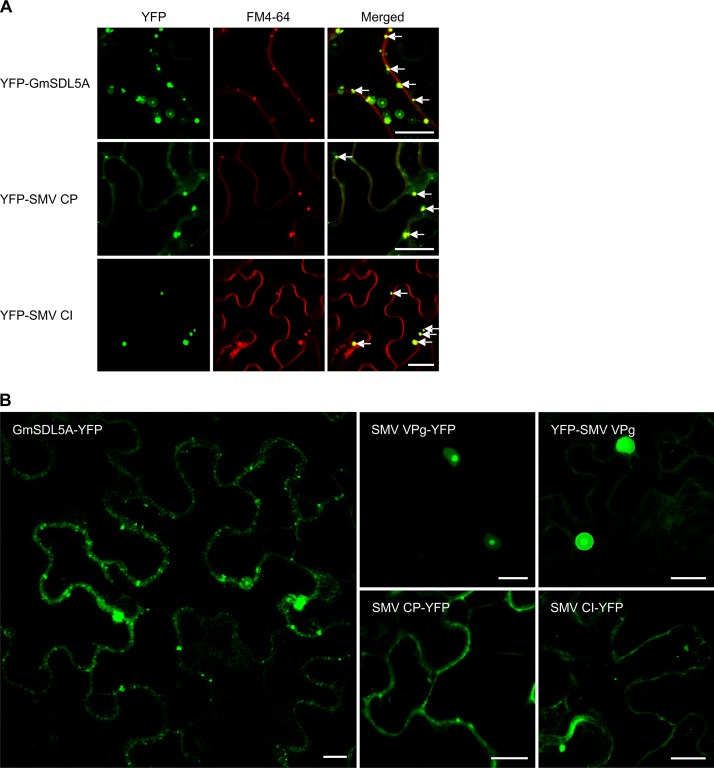
Subcellular localization of GmSDL5A and SMV proteins in *N. benthamiana* cells. (A) Colocalization assay of GmSDL5A, SMV CP, or SMV CI fused with a YFP tag at the N terminus, with FM4-64 staining. White arrows indicate the colocalization of the GmSDL5A or SMV proteins with the endosome compartments labeled by FM4-64. (B) Subcellular localization assay of GmSDL5A and SMV proteins fused with a YFP tag at the C terminus. Scale bar = 20 μm.

Bimolecular fluorescence complementation (BiFC) assays were conducted with Nicotiana benthamiana leaf cells to confirm the interactions of GmSDL5A with SMV CP, VPg, and CI. Indeed, positive interactions were found between GmSDL5A and the three SMV proteins (Fig. 1C and D). The GmSDL5A and SMV CP interactions took place on the PM, as revealed by the colocalization with FM4-64, a PM-staining dye (Fig. 1C). In contrast, the interaction of GmSDL5A with either VPg or CI occurred in the cytoplasm near the PM. As FM4-64 is a styryl dye that can also be used to monitor endocytosis and endosome localization (24), our data indicated that the interacting complex of GmSDL5A with VPg or CI was present in the endosomes labeled by FM4-64 (Fig. 1C). No positive interactions were found in the leaf cells coinfiltrated with the empty BiFC vector and GmSDL5A (Fig. 1D). Transient expression of GmSDL5A or SMV CP and CI proteins (with an N-terminal yellow fluorescent protein [YFP]) alone in N. benthamiana leaf cells stained by FM4-64 showed that GmSDL5A, CP, or CI subcellularly localized to the endosomes labeled by FM4-64 (Fig. 2A). They showed similar subcellular distribution patterns when YFP was tagged to their C termini (Fig. 2B). When expressed alone, VPg predominantly targeted the nucleus (Fig. 2B).

### Knockdown of *GmSDL5A* and *GmSDL12A* in soybean suppresses SMV infection.

**FIG 3:**
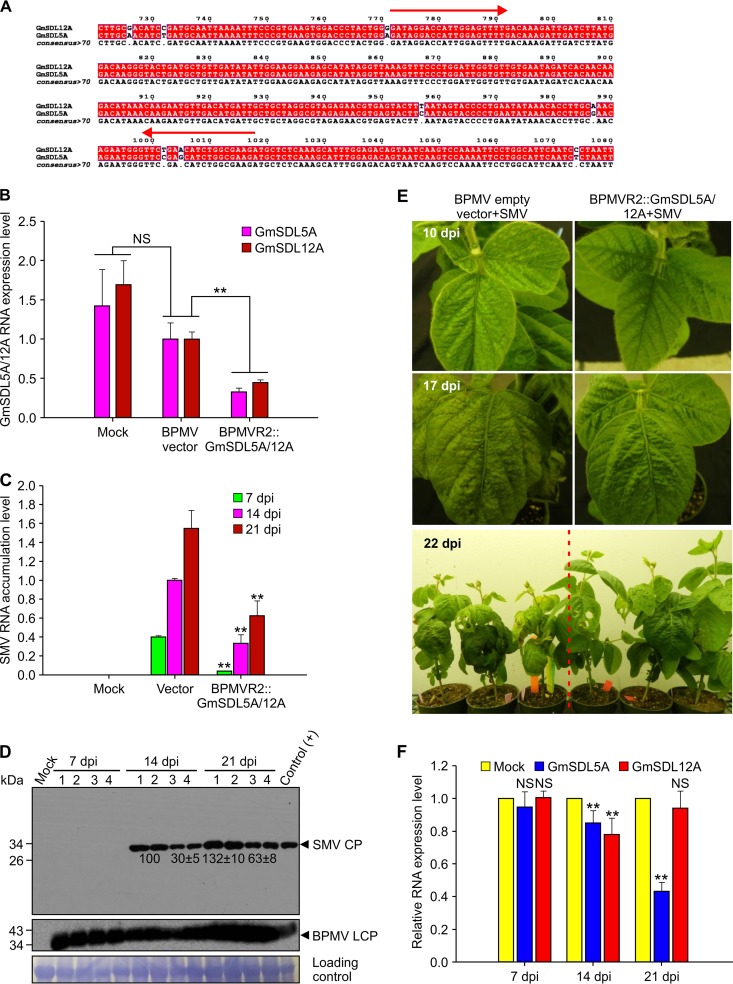
Effect of silencing of *GmSDL5A* and *GmSDL12A* on SMV accumulation and symptom development in soybean cultivar Williams 82 plants. (A) Alignment of partial nucleotide sequences between *GmSDL5A* and *GmSDL12A*. The conserved region between two red arrows was inserted into the BPMVR2M vector for gene silencing. (B) Gene silencing efficiency of *GmSDL5A* and *GmSDL12A* in soybean plants was analyzed by qRT-PCR at 21 days postbombardment. (C) qRT-PCR analyses of the accumulated SMV RNA level in the control and *GmSDL5A*- and *GmSDL12A*-silenced plants at 7, 14, and 21 days postinfection (dpi) with SMV. The soybean ATP-binding cassette transporter (ABCT) gene was used as an internal control. Data are means with standard deviations (SD) of results from three biological replicates. **, *P < *0.01 (very significant). (D) Western blot analyses of the accumulated SMV CP protein level in the control and *GmSDL5A*- and *GmSDL12A*-silenced plants at 7, 14, and 21 dpi. Lanes 1 and 2, samples from the BPMV empty vector control and SMV-L infectious-clone-coinfected systemic leaves; lanes 3 and 4, samples from BPMVR2::GmSDL5A/12A and SMV-L infectious-clone-coinfected systemic leaves. The relative intensities of the bands of the SMV CP protein levels were quantified by ImageJ software. (E) Symptom development in the control and *GmSDL5A*- and *GmSDL12A*-silenced plants at 10, 17, and 22 dpi. (F) qRT-PCR analysis of the mRNA levels of *GmSDL5A* and *GmSDL12A* in SMV-infected soybean plants at 7, 14, and 21 dpi. The soybean ATP-binding cassette transporter (*ABCT*) gene was used as an internal control. Data are means with SD from three biological replicates. **, *P* < 0.01; NS, not significant.

To investigate whether GmSDL5A is essential for SMV infection, we used an improved bean pod mottle virus (BPMV)-based virus-induced gene silencing (VIGS) vector (25, 26) to knock down *GmSDL5A* expression in soybean. Since GmSDL5A and GmSDL12A are highly similar (>98% identity) at both the amino acid and nucleotide levels and, thus, are likely functionally redundant, we generated a vector named BPMVR2::GmSDL5A/12A to target the conserved nucleotide sequence region shared between them (Fig. 3A). This modified VIGS vector was expected to simultaneously knock down both *GmSDL5A* and *GmSDL12A*. Soybean plants (Glycine max cv. Williams 82) were bombarded with the empty BPMV vector or BPMVR2::GmSDL5A/12A constructs. At 14 days postbombardment (dpb), treated plants showed typical symptoms induced by BPMV, and the presence of BPMV was confirmed by reverse transcription-PCR (RT-PCR) and Western blotting. We evaluated gene silencing efficacy in these plants by quantitative RT-PCR (qRT-PCR) (Fig. 3B). We found that at 21 dpb, the expression levels of *GmSDL5A* and *GmSDL12A* were significantly reduced (∼70% for *GmSDL5A* and ∼60% for *GmSDL12A*) in the soybean plants bombarded with BPMVR2::GmSDL5A/12A, in comparison with the control plants bombarded with the BPMV empty vector (Fig. 3B). Then, both *GmSDL5A*- and *GmSDL12A*-silenced and control plants were challenged with the SMV-L isolate (27). SMV infection was monitored at both the RNA and protein levels at 7, 14, and 21 days postinoculation (dpi). At all three time points, the SMV RNA level in the new upper leaves of *GmSDL5A*- and *GmSDL12A*-silenced soybean plants was significantly lower than that in the control plant treated with the BPMV empty vector (Fig. 3C). SMV CP protein was not detectable in any of the plants tested at 7 dpi. At 14 and 21 dpi, the SMV CP protein levels were substantially reduced in the *GmSDL5A*- and *GmSDL12A*-silenced plants compared to those in the control plants (Fig. 3D). The symptoms shown by the *GmSDL5A*- and *GmSDL12A*-silenced plants were also much milder than those in the control plants at 10, 17, and 22 dpi (Fig. 3E). In addition, we also investigated whether SMV infection affects the mRNA level of *GmSDL5A* and *GmSDL12A* at 7, 14, and 21 dpi in soybean plants. No significant difference was found between SMV-infected and control plants at 7 dpi (Fig. 3F). However, the expression levels of *GmSDL5A* were reduced in SMV-infected soybean plants at 14 and 21 dpi, and *GmSDL12A* was also downregulated at 14 dpi (Fig. 3F). These data suggest that GmSDL5A/12A is required for SMV infection and that downregulation of *GmSDL5A* and *GmSDL12A* might be part of induced resistance in response to SMV infection in soybean.

### GmSDL5A/12A homologs in *Arabidopsis* are essential for TuMV infection.

**FIG 4:**
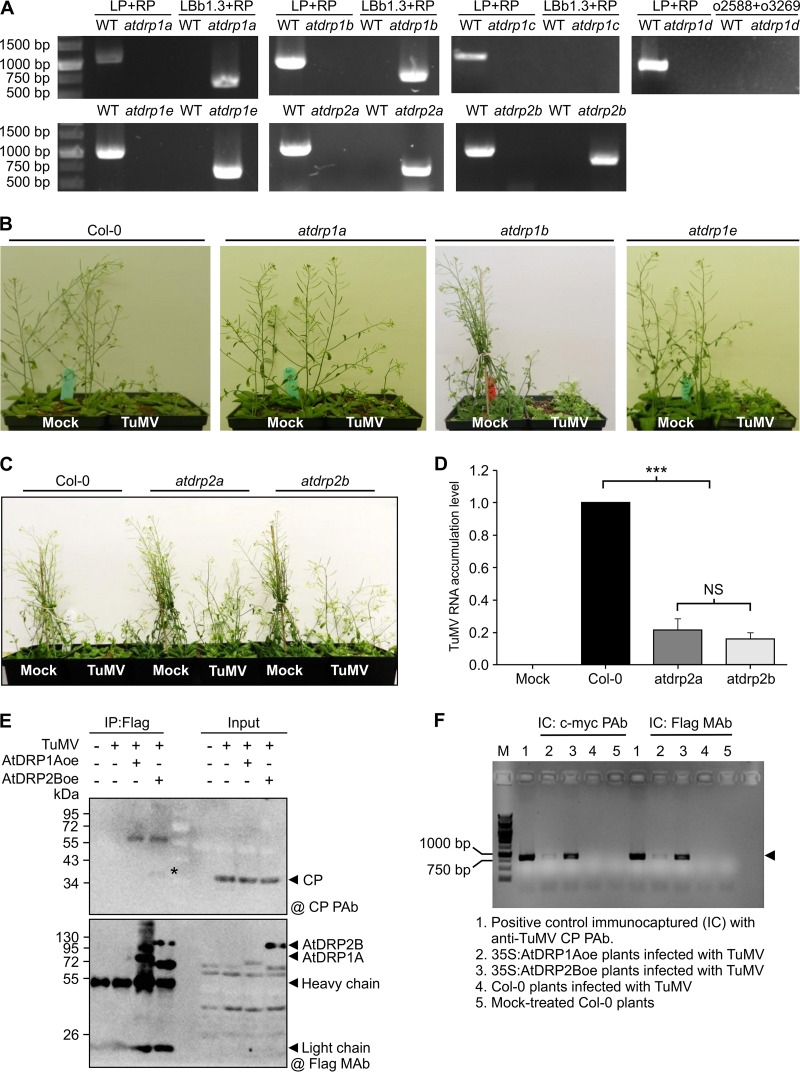
AtDRP2 interacts with TuMV virions, and knockout of *AtDRP2* in *Arabidopsis* confers resistance to TuMV infection. (A) PCR genotyping of *Arabidopsis* T-DNA mutants used in this study. (B) Phenotype of wild-type (WT) Col-0 and *atdrp1a*, *atdrp1b*, and *atdrp1e* mutants inoculated with TuMV or subjected to mock treatment at 16 dpi. (C) Phenotype of wild-type Col-0 and the *atdrp2a* and *atdrp2b* mutants inoculated with TuMV or subjected to mock treatment at 20 dpi. Note that both the *atdrp2a* and *atdrp2b* mutants showed much milder symptoms than those of wild-type Col-0 plants. (D) qRT-PCR analysis of TuMV RNA level in wild-type Col-0 and the *atdrp2a* and *atdrp2b* mutants at 16 dpi. The accumulation level of TuMV RNA in Col-0 was set to 1. Data are means with SD of results from three biological replicates. ***, *P < *0.01 (very significant); NS, not significant. (E) Co-IP assay for the presence of TuMV CP in the AtDRP1A or AtDRP2B protein complex. *Arabidopsis* transgenic overexpression plants of AtDRP1A or AtDRP2B fused with a flag-4× *myc* tag and wild-type Col-0 plants were inoculated with TuMV. The infected tissues were homogenized and immunocaptured with anti-flag M2 gel. The presence of TuMV CP in the AtDRP1A–flag-4× *myc* or AtDRP2B–flag-4× *myc* protein complexes was detected by immunoblotting using anti-TuMV CP PAb (@ CP PAb), and the detected protein is indicated with a black asterisk. (F) The presence of TuMV genomic RNA in the TuMV virions immunocaptured with anti-c-*myc* PAb or anti-flag MAb (@ Glag MAb) from wild-type Col-0 or transgenic *Arabidopsis* plants was detected by IC–RT-PCR. The arrowhead points to the amplified DNA fragment of TuMV RNA.

Since soybean is recalcitrant to genetic transformation and the soybean genome is a partially diploidized tetraploid, we switched to the *Arabidopsis*-TuMV pathosystem to further understand the role of dynamin in potyviral infection. BLAST searches against the NCBI database identified AtDRP1A (AT5G42080), which shares the highest amino acid similarity (86%) with GmSDL5A/12A in the *Arabidopsis* genome. As indicated in the introduction, AtDRP1A belongs to the AtDRP1 subfamily, which consists of five homologs (AtDRP1A to -E). Since AtDRP1 and AtDRP2 have almost the same function and share three conserved domains (15), the only two members (AtDRP2A and AtDRP2B) of the AtDRP2 subfamily were investigated in this study. To examine whether TuMV infection requires AtDRP1 and AtDRP2, we obtained *AtDRP1* and *AtDRP2* transfer DNA (T-DNA) lines from the *Arabidopsis* Biological Resource Centre (ABRC). These lines included SALK_061139, SALK_069077, and CS16362 for *atdrp1a* (28), CS401637 for *atdrp1b*, SALK_085100 for *atdrp1c*, SALK_043327 for *atdrp1d*, SALK_060080 for *atdrp1e* (29), SALK_071036 for *atdrp2a* (30, 31), and SALK_134887C (30, 31) and SALK_150606 for *atdrp2b* (31). We further performed PCR-based genotyping (32, 33) and obtained homozygous knockout mutants except for the *AtDRP1C* and *AtDRP1D* lines, in which no T-DNA insertion was detected (Fig. 4A). All these *atdrp* mutants showed growth and developmental phenotypes very similar to those of wild-type *Arabidopsis* plants. The *Arabidopsis* mutants and wild-type plants were mechanically inoculated with TuMV inoculum sampled from N. benthamiana leaves infected with an infectious pCambiaTuMV::GFP clone (32). As expected, the wild-type plants showed typical symptoms caused by TuMV infection (32, 33). Single *atdrp1* mutants (*atdrp1a*, *atdrp1b*, and *atdrp1e* mutants) displayed similar symptoms (Fig. 4B) and accumulated similar amounts of viral RNA (data not shown). However, homozygous *atdrp2* mutants (the SALK_071036 line for *atdrp2a* and the SALK_134887C line for *atdrp2b*) showed much milder symptoms than wild-type plants (Fig. 4C). The viral RNA level in both *atdrp2* mutants was reduced by more than 80% at 16 dpi (Fig. 4D). These results suggest that knockout of either *AtDRP2A* or *AtDRP2B* inhibits TuMV infection.

To further investigate whether AtDRP1 or AtDRP2 is associated with the TuMV virions, we generated transgenic *Arabidopsis* plants (in the ecotype Col-0 background) expressing AtDRP1A or AtDRP2B fused with a flag-4× c-*myc* tag and then inoculated these transgenic plants with TuMV-green fluorescent protein (GFP). The protein fusion AtDRP1A–flag-4× c-*myc* or AtDRP2B–flag-4× c-*myc* (with their respective protein complexes) in TuMV-infected tissues was subjected to coimmunoprecipitation (co-IP) with anti-flag M2 gel and detected by immunoblotting. We found that TuMV CP was coimmunoprecipitated with AtDRP2B–flag-4× c-*myc* but not with AtDRP1A–flag-4× c-*myc* (Fig. 4E), suggesting that the TuMV CP is associated with AtDRP2B. To confirm this result, we further performed an immunocapture (IC)–RT-PCR assay (34). Transgenic AtDRP2B–flag-4× c-*myc* or AtDRP1A–flag-4× c-*myc* plants infected by TuMV were sampled and captured by anti-myc polyclonal antibody (PAb) or anti-flag monoclonal antibody (MAb), and the purified complexes were analyzed by using RT-PCR to detect the viral RNA. Consistently, the viral genomic RNA was detected in the sample immunocaptured from the AtDRP2B–flag-4× c-*myc* transgenic plants infected by TuMV (Fig. 4F). A trace amount of TuMV viral RNA was also detected in the sample immunocaptured from the transgenic AtDRP1A–flag-4× c-*myc Arabidopsis* plants infected by TuMV. As AtDRP2 showed much greater affinity to TuMV virions than AtDRP1 and is essential for TuMV infection, we thus focused on AtDRP2.

### Overexpression of AtDRP2 promotes TuMV infection.

**FIG 5:**
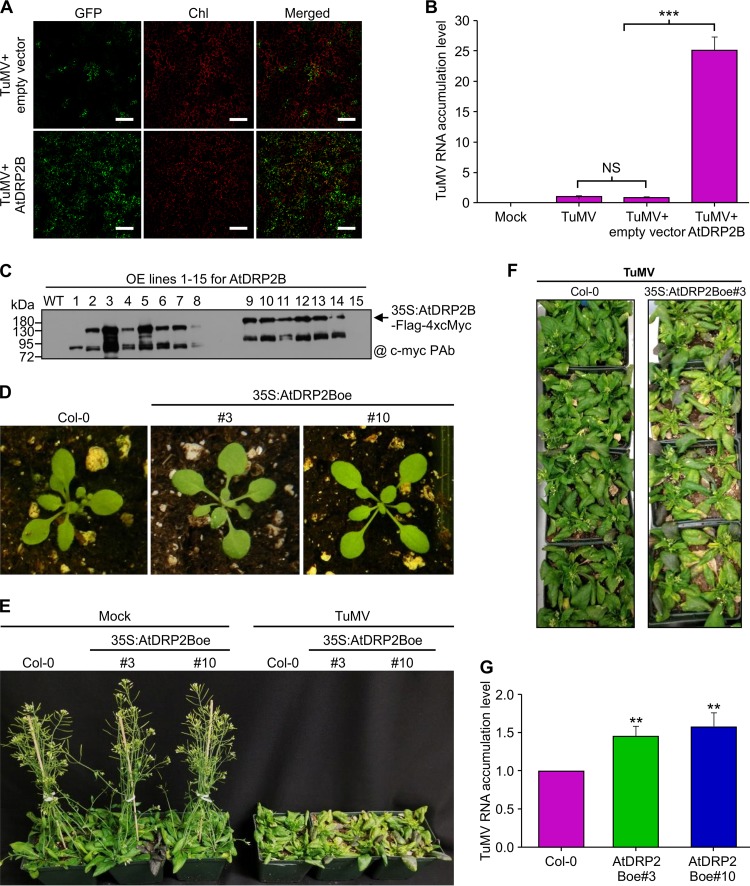
Overexpression of AtDRP2 promotes TuMV RNA accumulation and symptom development. (A) Transient overexpression of AtDRP2B promotes TuMV accumulation in epidermal cells of *Nicotiana benthamiana* leaves. Fluorescent intensities of TuMV::6K2-GFP under different treatments were measured at 3 dpa. Scale bar = 300 μm. (B) qRT-PCR analysis of TuMV genomic RNA levels under different treatments at 2 dpa. ***, *P < *0.01 (very significant); NS, not significant. Primers specific for the cDNA fragment coding for TuMV CP were used. (C) Verification of T0 *Arabidopsis* lines expressing AtDRP2B by Western blotting. OE, overexpression. (D) Wild-type Col-0 and transgenic *Arabidopsis* seedlings under normal growth conditions. #3, 35S:AtDRP2Boe#3; #10, 35S:AtDRP2Boe#10. (E) Wild-type and transgenic AtDRP2B *Arabidopsis* plants infected by TuMV or mock infected at 16 dpi. (F) Top view of the wild-type and 35S:AtDRP2Boe#3 *Arabidopsis* plants infected by TuMV at 16 dpi. (G) qRT-PCR analysis of the TuMV RNA level in wild-type and transgenic AtDRP2B *Arabidopsis* plants at 18 dpi. Data are means of results from three biological replicates, with SD. **, *P < *0.01; NS, not significant.

We investigated the effect of transient overexpression of AtDRP2 on TuMV accumulation. N. benthamiana leaves were coinfiltrated with a TuMV full-length infectious clone, pCambiaTuMV::6K2-GFP, together with an AtDRP2B expression construct or an empty vector as a control. We found that coexpression of AtDRP2B remarkably enhanced the fluorescent intensities of GFP directly resulting from TuMV infection (Fig. 5A). Virus accumulation was assessed in infiltrated leaves at 2 days postagroinfiltration (dpa) by qRT-PCR. Consistently, we found a significant increase (>20-fold) of TuMV genomic RNA when plants were coinfiltrated with AtDRP2B compared to that of control plants (Fig. 5B). To further analyze the effect of AtDRP2 on TuMV infection, transgenic *Arabidopsis* lines overexpressing AtDRP2B were generated. A total of 15 independent T0-positive lines were obtained and the overexpression of AtDRP2B in these lines was identified by immunoblotting (Fig. 5C). Two independent T2 lines (35S:AtDRP2Boe#3 and 35S:AtDRP2Boe#10) (Fig. 5D) were challenged with TuMV-GFP. In comparison with the wild-type plants, the transgenic plants overexpressing AtDRP2B were more susceptible to TuMV infection and developed more-severe symptoms (yellowish and dwarf phenotypes) (Fig. 5E and F). Consistently, higher levels of the viral genomic RNA were also detected in the AtDRP2B overexpression plants (Fig. 5G). These data support the possibility that AtDRP2 positively regulates TuMV infection.

### AtDRP2 interacts with TuMV 6K2, VPg, CP, and CI.

**FIG 6:**
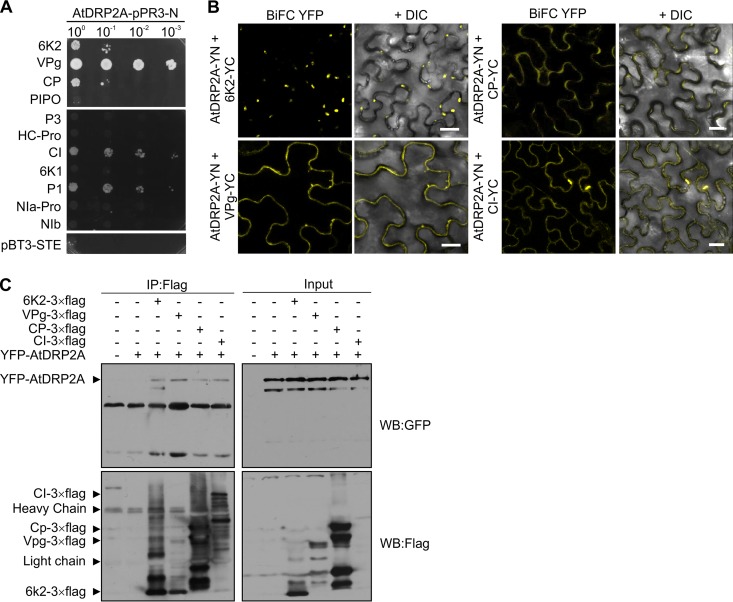
AtDRP2 interacts with TuMV proteins. (A) Protein-protein interaction assay between AtDRP2A and each of 11 TuMV-encoded proteins by using a membrane yeast two-hybrid method. (B) Positive interactions between AtDRP2A and each of four TuMV proteins, 6K2, VPg, CP, and CI, in *N. benthamiana* cells confirmed by BiFC assay. The YFP field and overlay of YFP with differential interference contrast (DIC) fields are shown. Scale bar = 20 μm. (C) AtDRP2A can form complexes with TuMV 6K2, VPg, CP, or CI in *N. benthamiana* cells as revealed by co-IP assay. Different cell lysates were immunoprecipitated with anti-Flag M2 beads, separated by SDS-PAGE, and immunoblotted with anti-Flag or anti-GFP IgG. WB, Western blot.

**FIG 7:**
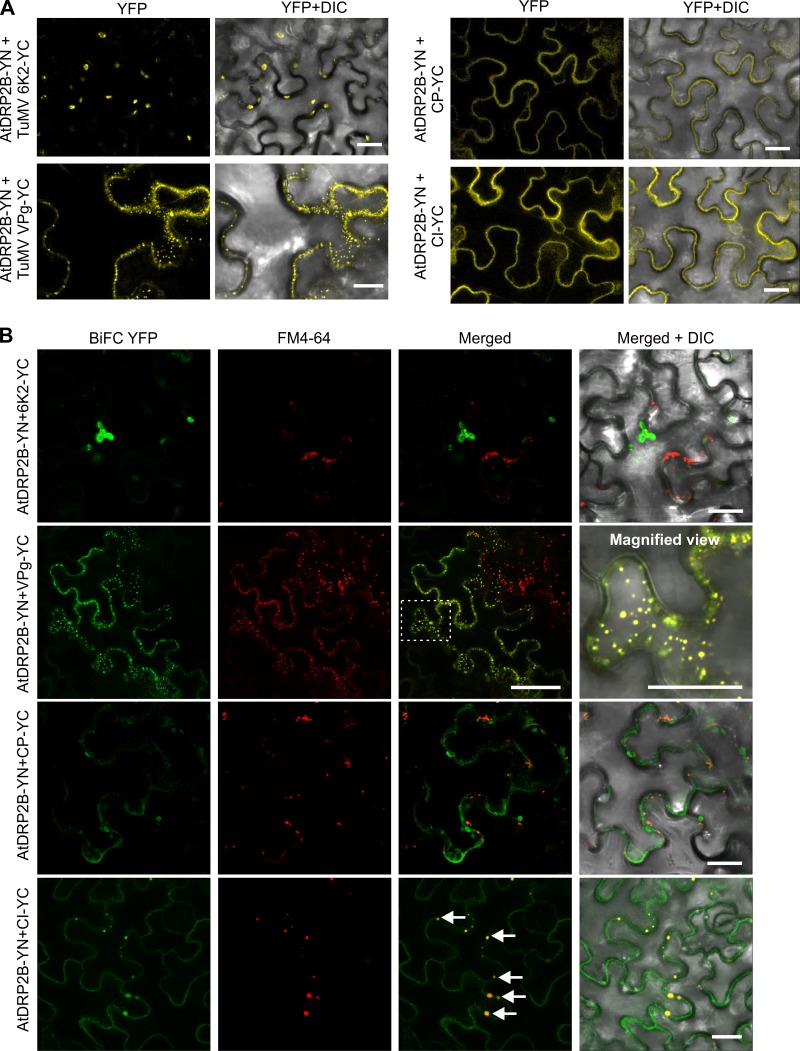
TuMV proteins VPg and CI localize in PM-derived endosomes. (A) BiFC assay of the interaction of AtDRP2B with TuMV proteins 6K2, VPg, CP, and CI in *N. benthamiana* cells. Scale bar = 20 μm. (B) Colocalization of the endocytosis tracing marker FM4-64 with the YFP signals resulting from the interaction between each of the four TuMV proteins (6K2, VPg, CP, and CI) with AtDRP2B. The area in the white rectangle is magnified to show the colocalization signals of the interaction complex of TuMV VPg and AtDP2B with FM4-64. Arrows point to the interaction complex of TuMV CI and AtDRP2B labeled by FM4-64 staining.

We then investigated whether AtDRP2 interacts with TuMV proteins by conducting an mYTH assay. Among the 11 viral proteins, 6K2, VPg, CP, CI, and P1 showed a positive interaction with AtDRP2A (Fig. 6A). BiFC assays were further carried out to confirm these interactions in N. benthamiana. Confocal microscopy detected positive signals in tissues coexpressing AtDRP2A and the four viral proteins, including 6K2, VPg, CP, and CI (Fig. 6B). No positive interaction signals were detectable between AtDRP2A and seven other viral proteins (data not shown). Similar results were also found for AtDRP2B (Fig. 7A). Interestingly, TuMV 6K2 interacted with either AtDRP2A or AtDRP2B in the cytoplasm and formed ring-like structures (Fig. 6B and 7A). The interaction of AtDRP2A or AtDRP2B with the three remaining viral proteins apparently led to the formation of numerous punctate structures in proximity to the PM (Fig. 6B and 7A).

To further confirm the protein-protein interactions, we conducted a co-IP assay. TuMV viral proteins were fused with a 3× flag tag, and AtDRP2A was tagged by an N-terminal YFP. These fusions were transiently coexpressed in N. benthamiana leaves, followed by co-IP. As shown in Fig. 6C, the four TuMV proteins 6K2, VPg, CP, and CI could be immunoprecipitated with the antibodies against YFP-AtDRP2A. Taken together, these data suggest that AtDRP2A or AtDRP2B interacts with TuMV 6K2, VPg, CP, and CI *in vivo*.

### TuMV VPg and CI are associated with endosomes.

As the punctate structures resulting from interactions of AtDRP2A or AtDRP2B with the viral proteins VPg, CI, and CP near the PM may be involved in endocytosis, we examined this possibility using a styryl dye, FM4-64, which is an established marker for PM internalization to localize endocytosis and endosomes (24). *N. benthamiana* leaves coexpressing AtDRP2B-YN and each of the four viral proteins fused with YC, e.g., 6K2-YC, VPg-YC, CP-YC, and CI-YC, were stained with FM4-64. As shown in Fig. 7B, the punctate structures labeled by the interaction complex of AtDRP2B and VPg or CI were stained by FM4-64, and many of the AtDRP2B/VPg colocalization signals were on or near the PM. At higher magnification, some of the relatively larger VPg-containing punctate structures were distant from the PM (Fig. 7B, right panel of the second row). Almost all the AtDRP2B/CI-containing punctate structures were on the PM. In the case of the AtDRP2B and 6K2 complex or AtDRP2B and CP complex, the BiFC signals did not colocalize with FM4-64 staining (Fig. 7B). These data indicate that TuMV VPg and CI, but not 6K2 and CP, are associated with the endosomes.

### AtDRP2 is recruited to the 6K2-containing VRC in TuMV-infected cells.

**FIG 8:**
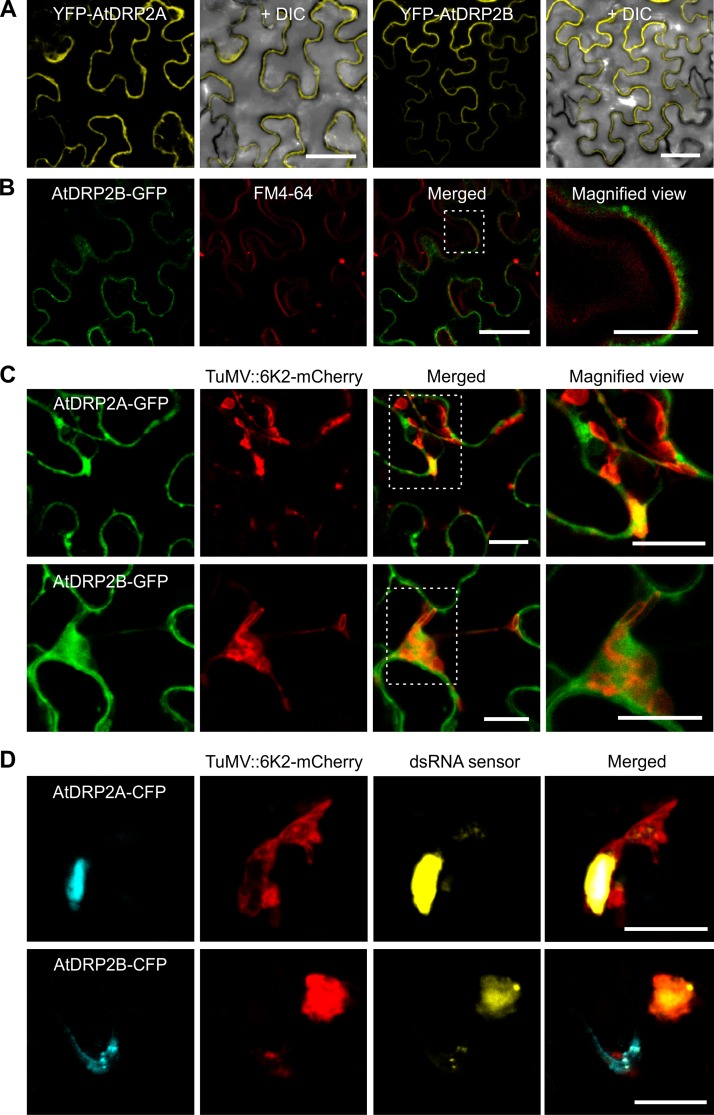
AtDRP2 colocalizes with TuMV VRC in *N. benthamiana* cells. (A) Subcellular localization of AtDRP2A or AtDRP2B fused with an YFP tag at the N terminus. (B) Colocalization of AtDRP2B fused with a C-terminal GFP tag and a PM marker, FM4-64. (C) Colocalization of AtDRP2A or AtDRP2B fused with a C-terminal GFP tag and 6K2-mCherry (from the recombinant virus TuMV::6K2-mCherry). Micrographs were taken at 72 hpa. (D) Colocalization of AtDRP2A or AtDRP2B fused with a C-terminal CFP tag, 6K2-mCherry (from TuMV::6K2-mCherry) and dsRNA (yellow). Scale bar = 20 μm.

Since AtDRP2 behaves like a host factor of TuMV, we checked the subcellular localization of AtDRP2A and AtDRP2B. Regardless of the fluorescent protein fused to their N or C terminus, AtDRP2A and AtDRP2B were apparently distributed in the cytoplasm along with the PM (Fig. 8A). Further costaining with FM4-64 revealed that AtDRP2 did not exactly overlap but was in proximity to the FM4-64 staining (Fig. 8B).

To investigate whether AtDRP2 is recruited by the VRC for TuMV infection, we coexpressed AtDRP2A or AtDRP2B in *N. benthamiana* leaf cells infected by a recombinant TuMV, pCambiaTuMV::6K2-mCherry. The modified virus contains an extra copy of 6K2 tagged by mCherry, so 6K2-containing VRCs may be visualized by confocal microscopy (35). We found that AtDRP2 was associated or colocalized with 6K2-containing VRCs during TuMV infection (Fig. 8C). In addition, when a double-stranded RNA (dsRNA) sensor (36) was coexpressed with AtDRP2A or AtDRP2B tagged by CFP in the leaf cells infected by pCambiaTuMV::6K2-mCherry, dsRNA signals were colocalized with the VRCs containing mCherry-tagged 6K2 and CFP-tagged AtDRP2 (Fig. 8D). These results suggest that AtDRP2 is recruited by TuMV VRCs.

### Treatment of a chemical inhibitor of endocytosis suppresses TuMV replication and intercellular movement.

**FIG 9:**
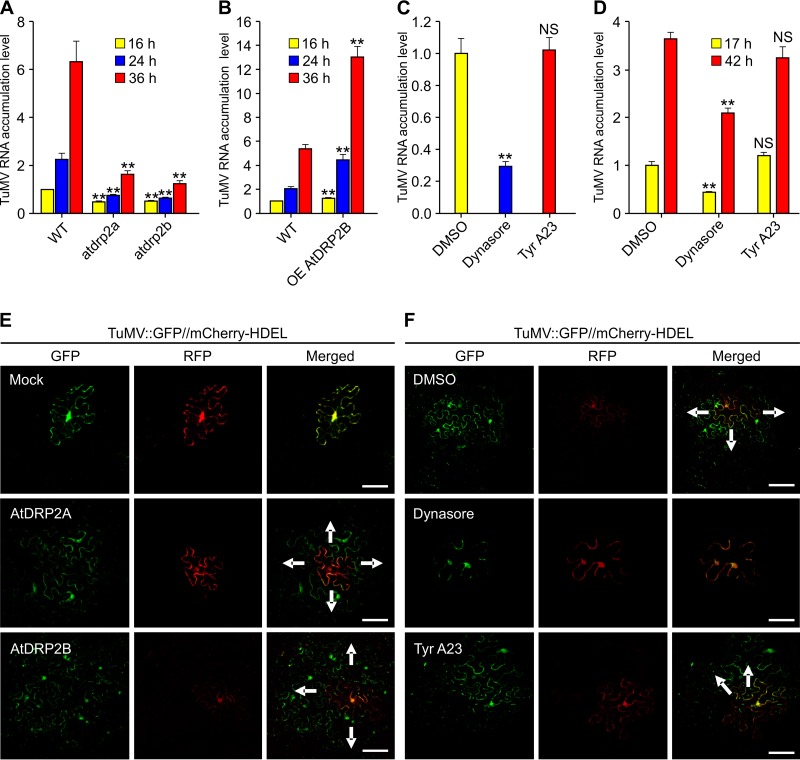
TuMV infection is positively regulated by AtDRP2 but suppressed by a chemical inhibitor of endocytosis. (A, B) TuMV infection assay of protoplasts of *Arabidopsis atdrp2* knockout mutants (A) and transgenic AtDRP2 overexpression plants (B). The accumulation level of TuMV genomic RNA in wild-type *Arabidopsis* was set to 1. Data are means with SD from two biological replicates. **, *P < *0.01 (very significant). (C) Dynasore and Tyr A23 treatments to determine TuMV RNA accumulation in *N. benthamiana* cells. Agrobacterial cultures containing PCB301-TuMV-GFP//mCherry-HDEL (OD at 600 nm [OD_600_], 0.2) were coinfiltrated with either DMSO, 50 µM dynasore, or 150 µM Tyr A23, and inoculated leaves were harvested at 58 hpa for qRT-PCR assay. *mCherry* transcripts were used as an internal control. **, *P < *0.01 (very significant); NS, not significant. (D) The effect of dynasore and Tyr A23 treatments on TuMV RNA replication in *Arabidopsis* protoplasts. *Arabidopsis* protoplasts transfected with pCambiaTuMV::GFP were treated with DMSO, 100 µM dynasore, or 150 µM Tyr A23, and TuMV RNA levels were quantified by qRT-PCR at 17 and 42 hpt. *actin II* transcripts were used as an internal control. **, *P  < *0.01 (very significant); NS, not significant. (E, F) Overexpression of AtDRP2 (E) or chemical inhibitor treatments (F) on TuMV in an intercellular-movement assay of *N. benthamiana* epidermal cells. PCB301TuMV-GFP//mCherry-HDEL (OD_600_, 0.001) was agroinfiltrated with either buffer, AtDRP2A–flag-4× *myc*, or AtDRP2B–flag-4× *myc* (E) or DMSO, 50 µM dynasore, or 150 µM Tyr A23 (F). The images were taken at 72 hpa (E) or 92 hpa (F). Green and red fluorescence resulted from the translation of the GFP-tagged recombinant TuMV and the mCherry-HDEL expression cassette, respectively. Cells labeled by double fluorescence indicate primarily infected cells; secondarily infected cells emit only green fluorescence. Arrows point to the direction of viral movement. Scale bars = 100 µm.

To confirm the possible role of AtDRP2 in viral replication, we conducted protoplast transfection assays. Protoplasts were isolated from wild-type *Arabidopsis*, *atdrp2a* and *atdrp2b* mutants, and AtRDP2 overexpression lines and then transfected with pCambiaTuMV::GFP (32), followed by qRT-PCR to monitor viral RNA accumulation at 16, 24, and 36 h posttransfection (hpt). TuMV viral RNA levels significantly decreased in both the *atdrp2a* and *atrdp2b* mutants, compared with those in the wild-type control, at all the three time points examined (Fig. 9A). In contrast, significantly higher levels of viral RNA were detected in the protoplasts from AtDRP2B overexpression transgenic lines (Fig. 9B).

To address the role of endocytosis in TuMV infection, we used two chemical inhibitors of endocytosis, dynasore and Tyrphostin A23 (Tyr A23) (37–39). Dynasore is known as a noncompetitive and reversible inhibitor which specifically targets dynamin to disrupt its GTPase activity (40), and Tyr A23 inhibits internalization of the transferrin receptor by perturbing the interaction between tyrosine motifs and the medium-chain subunit of the AP2 (41). *N. benthamiana* leaves were infiltrated with either Tyr A23, dynasore, or dimethyl sulfoxide (DMSO) and agrobacterial cultures containing PCB301TuMV-GFP//mCherry-HDEL. At 58 h postagroinfiltration (hpa), inoculated leaves were harvested to quantify the viral genomic RNA level by qRT-PCR. We found that treatment of dynasore significantly inhibited TuMV RNA accumulation (Fig. 9C). A similar result was also obtained from the TuMV RNA replication assay using *Arabidopsis* protoplasts (Fig. 9D). These data support the finding that endocytosis is involved in TuMV infection.

To further examine whether AtDRP2 affects viral intercellular movement, we introduced the vector PCB301TuMV-GFP//mCherry-HDEL (42) (using a low optical density [OD] of agrobacterial culture) into *N. benthamiana* leaves coexpressing AtDRP2A or AtDRP2B. This vector contains two expression cassettes, including one for the generation of a recombinant TuMV tagged by GFP and one for the expression of mCherry-HDEL. The primary-infection sites emit both red and green fluorescences, and the secondary-infection cells emit only green fluorescence. Therefore, this vector can be used to distinguish primary and secondary infections. In the control leaf cells infiltrated with PCB301TuMV-GFP//mCherry-HDEL and buffer (mock infection), the inoculated cells emitted GFP and RFP fluorescence at 3 dpa (Fig. 9E), indicating that viral intercellular movement did not occur at this time point. However, at the same time point, coexpression of either AtDRP2A or AtDRP2B facilitated the cell-to-cell movement of the recombinant virus into secondarily infected cells emitting green fluorescence only (Fig. 9E). We also conducted an inhibitor assay using the chemical effectors dynasore and Tyr A23 in *N. benthamiana*. At 92 hpa, in the DMSO- or Tyr A23-treated leaf cells, viral intercellular movement occurred, while in the dynasore-treated cells, no viral cell-to-cell movement occurred (Fig. 9F). Taken together, these results suggest that overexpression of AtDRP2 promotes both virus replication and intercellular movement, which can be suppressed by the endocytosis inhibitor dynasore.

### TuMV proteins VPg and CI are delivered into both the EE and the LE, which can be inhibited by the dynamin inhibitor dynasore.

**FIG 10:**
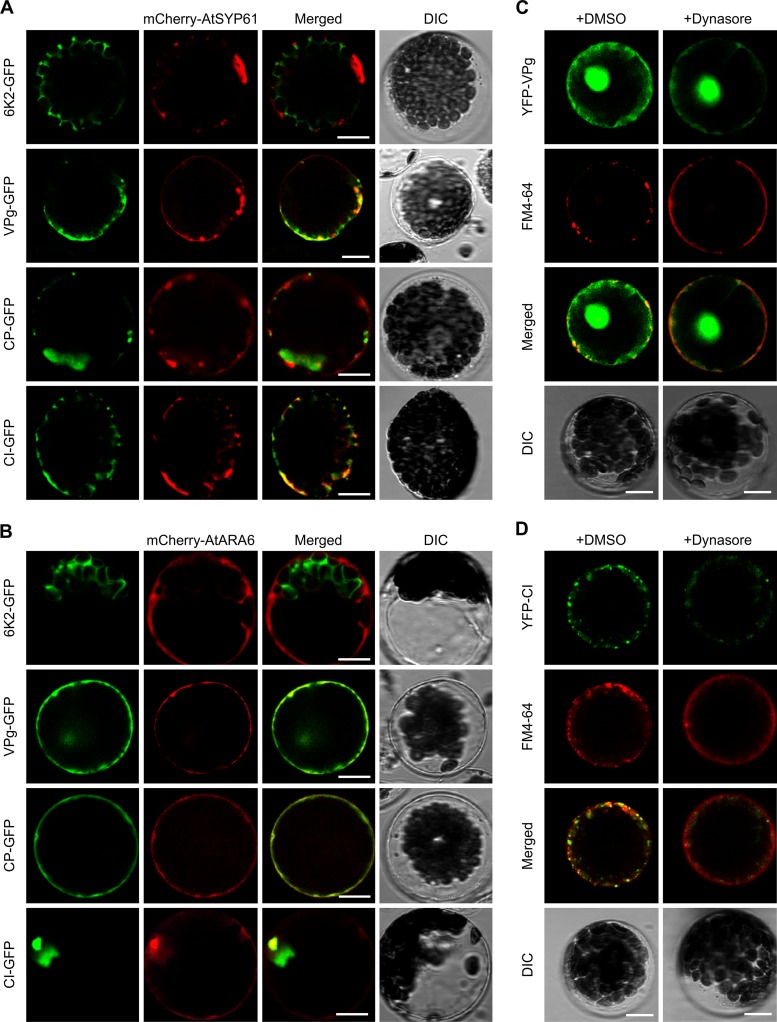
TuMV proteins VPg and CI localize to post-Golgi compartments. (A, B) Colocalization assay of TuMV fusion proteins with the early endosome marker mCherry-AtSYP61 (A) and the late endosome marker mCherry-AtARA6 (B) in *Arabidopsis* protoplasts. Images were taken at 20 hpt. (C, D) Effect of dynasore treatment on the subcellular localization of YFP-TuMV VPg (C) and YFP-TuMV CI (D). Protoplasts expressing YFP-TuMV VPg or YFP-TuMV CI at 16 hpt were treated with 100 µM dynasore or DMSO for 3 h and then 40 µM FM4-64 for 1 h. Scale bar = 10 μm.

To further characterize the endosomal vesicles labeled by the interaction complexes of AtDRP2 and viral proteins, we used fluorescently tagged AtSYP61 (mCherry-AtSYP61) (43) and AtARA6 (mCherry-AtARA6) (41) as the EE and LE markers. In *Arabidopsis* protoplasts, we observed that a fraction of the EE and LE were labeled by TuMV VPg and CI (Fig. 10A and B). Interestingly, CP was evident in the LE but not in the EE. Since the TuMV CP- and AtDPR2-interacting complex did not colocalize with the endosomal structures labeled by FM4-64 (Fig. 7B), TuMV CP might hijack a different pathway to reach the LE. TuMV 6K2-GFP did not colocalize with either the EE or the LE marker.

To further confirm that TuMV proteins VPg and CI traffic in the endocytic pathway, we conducted a pharmacological interference assay with dynasore. In transfected protoplasts, treatment with dynasore changed the distribution pattern of YFP-VPg and YFP-CI (Fig. 10C and D). Fewer FM4-64-labeled endosomes were visible upon dynasore treatment. YFP-VPg and YFP-CI were concentrated on the PM and rarely labeled with FM4-64, compared with what occurred with DMSO treatment. These results support the finding that TuMV proteins VPg and CI are delivered to the EE and LE via the endocytic pathway.

## DISCUSSION

Proteomic techniques are widely used to identify the protein components of macromolecular complexes that participate in different biological processes, and proteome-based studies on pathogenesis of several plant viruses have made important contributions to the elucidation of molecular plant-virus interactions (44). The use of nano-LC for the high-resolution separation of digested peptides in samples has greatly increased detection capacity, making it easy to analyze low-copy-number proteins. In this study, we used an LC-MS/MS method to identify the proteins associated with SMV virions purified in virus-infected soybean leaves. We detected two dominant proteins from purified SMV virions (Fig. 1). These two proteins showed a slight size difference but are the expected size of CP. Our top-down proteomics analysis confirmed that both bands correspond to the CP of SMV (Fig. 1; Table 1). The different migration rates of SMV CP might be due to nondetected phosphorylations, proteolysis, or partial degradation. This observation is consistent with those of a previous report on another potyvirus, PPV (45). In addition, we also detected SMV CI. Previous works suggest that, in addition to CP being a virion component, VPg, CI, and likely HC-Pro are physically associated with potyvirus virions (45–48). In this study, HC-Pro was not detected, possibly because only a small amount of HC-Pro was associated with SMV virions. We did not analyze the proteins with a molecular mass lower than that of the CP, which may explain why VPg was not detected in our purified virion samples.

We identified five novel host proteins associated with the virions (Table 1; see also Table S1 in the supplemental material). In addition to these proteins, other host proteins were present in small amounts in our purified virion preparations and are not listed here due to the conservative criteria employed in our study. The presence of these five host proteins could be attributed to copurification of cellular components during virion preparations or to purified virions sticking to cellular proteins. However, it is equally possible that some of these cellular proteins, like ribulose bisphosphate carboxylase, are present in virions merely because of their high abundance in the cytosol. Among the five identified soybean proteins, the dynamin protein GmSDL5A, which is involved in endocytosis and vesicle trafficking, was chosen for further study.

Dynamin participates in endocytosis in both plants and animals (13, 49). Since endocytosis is considered a fundamental process in biology, it is conceivable that endocytosis is involved in virus infection. In a previous study, clathrin, which is required in endocytic vesicle formation, was found to be associated with the human cytomegalovirus particles (50). More recently, components of endocytic and post-Golgi pathways have also been implicated in infection by several plant viruses (51–56). To explore whether GmSDL5A plays a role in SMV infection, we detected and confirmed that GmSDL5A interacted with several SMV proteins in yeast and *in planta* (Fig. 1). Interestingly, unlike TuMV P1, SMV P1 did not interact with GmSDL5A in yeast cells (Fig. 1, Fig. 6). It is well known that of the 11 known potyviral proteins, P1 is a hypervariable protein that is least conserved among potyviruses (17, 57). This may account for the different abilities of SMV P1 to bind to GmSDL5A and TuMV P1 to AtDRP2. In addition to GmSDL5A interacting with SMV VPg or CI, GmSDL5A alone localized to the endosome structures labeled by FM4-64, suggesting a role for GmSDL5A in the endocytosis of SMV proteins (Fig. 2). SMV CP or CI, but not SMV VPg, could enter into endosomal compartments in *N. benthamiana* cells (Fig. 2). To address the biological importance of GmSDL5A in SMV infection, we used a BPMV vector to knock down *GmSDL5A* and its highly conserved homologous gene *GmSDL12A* in soybean and found that knockdown of them significantly inhibits SMV infection (Fig. 3). Due to limitations of the soybean/SMV pathosystem, we used *Arabidopsis*/TuMV as a model to further investigate the role of dynamin proteins in potyvirus infection.

Computer-assisted analyses identified AtDRP1 and AtDRP2 in *Arabidopsis* that share high sequence identity with GmSDL5A. Logically, we checked whether knockout of *AtDRP1A* or *AtDRP2* affects TuMV infection. There are five isoforms of AtDRP1 and two isoforms of AtDRP2. None of the knockout mutants corresponding to these five *AtDRP1* genes displayed obviously different symptoms and accumulated significantly different levels of viral RNA after TuMV infection. However, knockout of either *AtDRP2A* or *AtDRP2B* significantly impaired TuMV infection (Fig. 4). Clearly, AtDRP2 plays important roles in TuMV infection. Since the double *atdrp2* mutant is gametophyte lethal (31), we cannot further evaluate the effect of a knockout of both *AtDRP2A* and *AtDRP2B* on TuMV infection. We thus, investigated the effect of overexpression of AtDRP2 on TuMV infection. We found that that either transient or transgenic overexpression of AtDRP2 promoted viral accumulation (Fig. 5). These data led us to suggest that AtDRP2 positively regulates TuMV infection.

To understand whether AtDRP2 affects virus infection through virus replication, intercellular movement, or both, we conducted protoplast transfection assays and confocal microscopy. We found that knockout of *AtDRP2A* or *AtDRP2B* in *Arabidopsis* inhibited viral RNA accumulation in protoplasts but that its overexpression enhanced viral RNA accumulation (Fig. 9A and B). Coexpression of either AtDRP2A or AtDRP2B also facilitated TuMV intercellular movement in *N. benthamiana* (Fig. 9E). Since more-robust viral replication is expected to accelerate viral intercellular movement, it is possible that the expedited spread of TuMV by the overexpression of AtDRP2 actually resulted from enhanced viral replication. Treatment with dynasore, a dynamin-specific chemical inhibitor, suppressed viral replication in *Arabidopsis* (Fig. 9D) and *N. benthamiana* (Fig. 9C) and intercellular movement in *N. benthamiana* (Fig. 9F). These data further support the assumption that AtDRP2 is a host factor that positively regulates virus replication (and possibly cell-to-cell movement) likely via endocytosis pathways.

To verify this assumption, we examined the effect of two chemical inhibitors of endocytosis, dynasore and Tyr A23, on TuMV infection. Treatment of dynasore indeed inhibited TuMV replication and intercellular movement significantly (Fig. 9D and F). As mentioned earlier, dynasore specifically targets dynamin to disrupt its GTPase activity and inhibits endocytosis (40). The antiviral role of dynasore corroborates the requirement of endocytosis and the dynamin-like proteins GmSDL5A and AtDRP2 in potyvirus infection. However, treatment of Tyr A23 did not affect TuMV replication and cell-to-cell movement (Fig. 9C, D and F). This is consistent with a previous report that Tyr A23 does not affect TuMV intercellular movement (58). As an endocytosis inhibitor, Tyr A23 inhibits the recruitment of endocytic cargo into clathrin-coated vesicles formed at the PM (41). These data suggest that dynamin may regulate viral infection independently of the clathrin-mediated endocytic pathway. Consistently, we also found that TuMV VPg and CI could enter into the EE and LE and that dynasore treatment inhibited their trafficking into endosomes, which further confirm the role of endocytosis of TuMV VPg and CI by AtDRP2 in TuMV infection.

To explore the molecular mechanism underlying the functional role of AtDRP2 in virus infection, we conducted several types of protein-protein interaction assays (mYTHS, BiFC, and co-IP) to screen for and confirm TuMV proteins that interact with AtDRP2. We found that AtDRP2 interacted with TuMV proteins 6K2, VPg, CP, and CI (Fig. 6). Since VPg, CP, and CI are all involved in virus movement, it is possible that AtDRP2 promotes virus cell-to-cell movement through its interaction with these viral proteins, particularly CI. It is well established that CI forms conical structures at the PD and is associated with viral particles (59). The facts that AtDRP2 is associated with virions (Fig. 4E and F) and the AtDRP2/CI interaction takes place along the PM (Fig. 7B) support this assumption. VPg and CI are multifunctional proteins, and both of them are the critical components of VRCs (17). Our confocal microscopy revealed that AtDRP2 was recruited to the VRC in TuMV-infected leaf cells (Fig. 8). Since virus replication requires remodelling of cellular members for the formation of membrane-bound VRCs and AtDRP2 plays an essential role in membrane remodelling and fusion, it is possible that the TuMV VRCs coopt AtDRP2 for VRC assembly. The exact mechanical role of AtDRP2 in the VRC remains to be further elucidated.

## MATERIALS AND METHODS

### Plant materials and growth conditions.

Soybean Williams 82 plants were used and grown in an insect-free growth chamber under a cycle of 16 h of light at 22°C and 8 h of darkness at 18°C. Plants were inoculated by biolistic bombardment with SMV infectious clone SMV-L (G2 strain) (60). Virus detection was carried out by enzyme-linked immunosorbent assay (ELISA) and RT-PCR as previously described (27).

All the *Arabidopsis* mutants used in this study were obtained from the *Arabidopsis* Biological Resource Centre. Homozygous plants of each line were screened as described previously (33). Transgenic *Arabidopsis* (ecotype Col-0) plants overexpressing AtDRP1A or AtDRP2B were obtained by the floral-dip method (61). Transformants were screened by direct spraying of solutions containing 20 mg/liter glufosinate-ammonium. Unless stated otherwise, all *Arabidopsis* plants were grown in a growth chamber with a 14 h photoperiod and a relative humidity of 75% at 23°C/21°C (light/dark). The *N. benthamiana* plants were grown in a growth room with a 16-h photoperiod and a relative humidity of 75% at 22°C.

### RNA extraction, synthesis of the first-strand cDNA, PCR, and qPCR.

Total RNA extraction from *G. max* cultivar Williams 82, *Arabidopsis*, and *N. benthamiana* tissues, synthesis of the first-strand cDNA, and PCR and qPCR were performed essentially as described previously (32, 62). Information on the primers used in this study is available upon request.

### Gene cloning and plasmid construction.

In this study, the Phusion high-fidelity DNA polymerase (New England Biolabs, USA) was used to amplify all DNA sequences, and Gateway technology (ThermoFisher Scientific, USA) was employed for plasmid construction. Coding sequences of GmSDL5A from soybean and AtDRP1A, AtDRP2A, and AtDRP2B from *A. thaliana* Columbia-0 were amplified from soybean or *Arabidopsis* cDNA as described above. Coding sequences of SMV and TuMV genes were amplified from the infectious clones SMV-L and pCambiaTuMV::GFP (60, 63), respectively. These cloned genes were recombined into pDONR221 prior to final recombination to plant expression vectors by LR reactions. AtDRP2A and AtDRP2B were recombined into pEarleyGate102, pEarleyGate103, and pEarleyGate104, giving C-terminal CFP, C-terminal GFP, and N-terminal YFP, respectively. pEarleyGate vectors are described in reference 64. For the BiFC assay, the SMV or TuMV genes and host genes were transferred into the modified pEarleyGate201-nYFP or pEarleyGate202-cYFP vector (65). The pBA-FLAG−4× Myc-DC vector (66) was used to yield a FLAG−4× Myc-tagged construct. All constructs were verified by sequencing to ensure that no errors were introduced by PCR amplification. All plant expression cassettes were electroporated into Agrobacterium tumefaciens strain GV3101. The GV3101 cells harboring the expression constructs were resuspended with the infiltration buffer and infiltrated into *N. benthamiana* leaves as previously described (67, 68).

### Virus-induced gene silencing assay in soybean.

To knock down the expression of GmSDL5A and GmSDL12A in soybean, a DNA-based BPMV-modified vector was used as previously reported (25, 26). Transcript levels of both GmSDL5A and GmSDL12A were evaluated by RT-qPCR. The soybean ATP-binding cassette transporter (ABCT) gene was used as an internal control (69).

### SMV virion purification.

For LC-MS/MS analysis, the SMV virions were prepared as previously reported, with some modifications (70). Briefly, 100 g systemically infected leaves of Glycine max were harvested 3 weeks postinoculation and homogenized in a Waring blender with chilled 0.5 M phosphate buffer, pH 7.0, containing 0.1% 2-mercaptoethanol and 0.01 M EDTA (4 ml of buffer for 1 g of leaf tissue). Homogenized tissue was filtered through two layers of cheesecloth, followed by low-speed centrifugation in a Beckman L8-80M ultracentrifuge (6,000 × *g* for 20 min) to remove plant debris. Clarified extracts were brought to 0.2 M NaCl containing Triton X-100 (0.1%, vol/vol), and then 6 g of polyethylene glycol with a molecular weight of 6,000 (PEG 6000) was added per 100 ml of extract. After the mixture was stirred for 0.5 h and incubated for 6 h, the precipitated virus was collected by low-speed centrifugation at 8,000 × *g* for 10 min. The precipitated virus was resuspended in 20 ml of 0.5 M phosphate buffer, pH 7.0, containing 0.1% 2-mercaptoethanol, 0.01 M EDTA, and 0.5 M urea, followed by low-speed centrifugation (8,000 × *g*, 10 min), and this step was repeated twice more. The virus was further concentrated by high-speed centrifugation for 2 h at 90,000 × *g* through a 10-ml cushion of 20% sucrose in an 80Ti rotor. Birefringence was checked by swirling the resuspended virus solution between two crossed polarizing prisms and observing the resulting light patterns. The virus-containing band was removed from the tube carefully and resuspended in 2 ml 0.1 M phosphate buffer, pH 8.0. After low-speed centrifugation at 10,000 × *g* for 5 min, the supernatant containing the purified virions was collected and stored long term at −80°C and short term at −20°C. Protein concentration was measured using NanoDrop 2000c spectrophotometers (Thermo Scientific).

### Protein separation and mass spectrometry.

Purified SMV virions were denatured by boiling the reaction mixture for 5 min and then loading it onto SDS-PAGE gels. The gels were then stained with a Coomassie brilliant blue solution (0.05% Coomassie brilliant blue G250, 50% methanol, 10% acetic acid) for 2 h and then destained (50% methanol, 10% acetic acid) for 5 h. The Coomassie blue-stained bands were excised from a gel rinsed 20 times in distilled water, destained, reduced, alkylated, and digested using the Thermo in-gel tryptic digestion kit (number 89871) according to the manufacturer’s protocol. The peptide digests were analyzed using an Easy-nLC 1000 nano system with a 75-µm by 15-cm Acclaim C_18_ PepMap column (Thermo Scientific) coupled to a Q Exactive Orbitrap mass spectrometer (ThermoFisher Scientific) (71). The flow rate was 300 nl min^−1^, and 10 µl of the protein digest was injected. The C_18_ column was equilibrated with 98% mobile phase A (water with 0.1% formic acid) and 2% mobile phase B (acetonitrile with 0.1% formic acid) and eluted with a linear gradient from 2% to 30% phase B over 18 min, followed by elution with a 30% to 98% gradient over 2 min, and then maintained for 10 min. The nanospray voltage was set at 2.1 kV, the capillary temperature was 275°C, and the S-lens RF level was 55. The Q-Exactive was operated in top 5 data-dependent acquisition mode with a full-scan mass range of 400 to 2,000 *m/z* at a resolution of 70,000, with an automatic gain control (AGC) of 1e^6^ and a maximum injection time (IT) of 250 ms. The MS/MS scans were acquired at a 17,500 resolution, an AGC of 2e^5^, a maximum IT of 50 ms, an intensity threshold of 8e^4^, a normalized collision energy of 27, and an isolation window of 1.2 *m/z*. Unassigned peptides and peptides that were singly charged and charged >4 times were not selected for MS/MS, and a 20-s dynamic exclusion was used. The Thermo .raw files were converted to .mgf using ProteoWizard v2, and the MS/MS scans were searched against the target/reverse UniProt Glycine max database and 11 proteins encoded by SMV (GenBank accession no. AEP04405.1) by using the X! Tandem search algorithm operated from the SearchGUI v.2.35 interface and processed in PeptideShaker v1.3.6. A 3-ppm precursor ion mass error and a 0.02-Da product ion error were used, along with carbamidomethylation as a constant modification and oxidation of methionine as a variable. A 1% false-discovery rate (FDR) was used at the protein, peptide, and peptide spectrum match levels.

### Confocal microscopy.

Confocal microscopy was performed by using a Leica TCS SP2 inverted confocal microscope (Leica Microsystems, Wetzlar, Germany) equipped with a 63× water-corrected objective in multitrack mode (72, 73). Collected images were analyzed with the Leica Application Suite for Advanced Fluorescence (LAS AF; version 2.35) software (Leica Microsystem).

### co-IP.

Coimmunoprecipitation (co-IP) experiments were performed as previously described, with some modifications (74). The virus genes were overexpressed in *N. benthamiana* leaves by using the modified high-expression vector pSK104 with a C-terminal 3× flag tag (75). The host genes were overexpressed in *N. benthamiana* leaves by using the pEarleyGate-104 vector with an N-terminal YFP tag. Immunoprecipitation was performed from *N. benthamiana* leaves at 2.5 days postinfiltration by using anti-Flag M2 affinity gel (Sigma, USA). Relevant antibodies were purchased from Sigma.

### Protoplast isolation and TuMV replication assay.

Mesophyll protoplasts were prepared from 4-week-old *Arabidopsis* leaves by the procedure described previously (76). About 1 × 10^5^ protoplasts were transfected with 30 µg pCambiaTuMV::GFP in 40% PEG 4000 in 0.8 M mannitol and 1 M CaCl_2_ at room temperature for 20 min. Transformed protoplasts were then washed and resuspended in W5 buffer (154 mM NaCl, 125 mM CaCl_2_, 5 mM KCl and 2 mM MES, pH 5.7) and incubated for the virus replication.

### FM4-64 staining and treatment of endocytosis chemical inhibitors.

*N. benthamiana* leaves were infiltrated with 40 µM FM4-64 (Invitrogen) and then transferred to the microscope for imaging.

The endocytosis chemical inhibitors dynasore (Sigma) and Tyr A23 (Sigma) were used to inhibit the endocytic pathway, as previously reported (37–39).

### Co-IP-derived virion binding assay.

A virion binding assay similar to that previously reported (77) was performed, with some modifications. Monoclonal antibody directed against the Flag tag was used as trapping antibodies to capture AtDR1A–4× c-*myc*-flag or AtDR2B–4× c-*myc*-flag and its binding complexes. TuMV-infected crude plant extract was added. Polyclonal rabbit antibodies against TuMV virions were used for virion detection.

### IC–RT-PCR assay.

Immunocapture (IC)–RT-PCR was performed to detect the binding virions with AtDRP1A or AtDRP2B as previously described (34).

### ImageJ analysis of data.

ImageJ was used to quantify average integrated density values of bands on immunoblots (78). The scanned images were saved in 16-bit .tiff format. The relative densities of the peaks with selected areas were calculated by the ImageJ software per the manual instructions.

### Sequence alignments and accession numbers.

Multiple sequence alignments of amino acid sequences were performed by using the MUSCLE algorithm online (http://www.ebi.ac.uk/Tools/msa/muscle/). Sequence data from this article can be found in SoyBase (https://soybase.org/) under accession numbers Glyma.08G023300 (GmSDL5A) and Glyma.05g217300 (GmSDL12A), in the *Arabidopsis* Information Resource (https://www.arabidopsis.org/) under accession numbers AT5G42080 (AtDRP1A), AT3G61760 (AtDRP1B), AT1G14830 (AtDRP1C), AT2G44590 (AtDRP1D), AT3G60190 (AtDRP1E), AT1G10290 (AtDRP2A), and AT1G59610 (AtDRP2B), and in GenBank under accession numbers EU871724.1 for SMV-L and EF028235.1 for TuMV.

## Supplementary Material

Supplemental file 1
